# Cellular electrophysiological effects of the citrus flavonoid hesperetin in dog and rabbit cardiac ventricular preparations

**DOI:** 10.1038/s41598-024-57828-y

**Published:** 2024-03-27

**Authors:** Aiman Saleh A. Mohammed, Gábor Mohácsi, Muhammad Naveed, János Prorok, Norbert Jost, László Virág, István Baczkó, Leila Topal, András Varró

**Affiliations:** 1 Department of Pharmacology and Pharmacotherapy, Albert Szent-Györgyi School of Medicine, University of Szeged, Szeged, Hungary; 2 HUN-REN-SZTE Research Group for Cardiovascular Pharmacology, Hungarian Research Network, Szeged, Hungary

**Keywords:** Hesperetin, Citrus flavonoids, Repolarization reserve, Proarrhythmia, Transmembrane ionic currents, Arrhythmias, Intracellular recording, Patch clamp

## Abstract

Recent experimental data shows that hesperetin, a citrus flavonoid, affects potassium channels and can prolong the QT_c_ interval in humans. Therefore, in the present study we investigated the effects of hesperetin on various transmembrane ionic currents and on ventricular action potentials. Transmembrane current measurements and action potential recordings were performed by patch-clamp and the conventional microelectrode techniques in dog and rabbit ventricular preparations. At 10 µM concentration hesperetin did not, however, at 30 µM significantly decreased the amplitude of the I_K1_, I_to_, I_Kr_ potassium currents. Hesperetin at 3–30 µM significantly and in a concentration-dependent manner reduced the amplitude of the I_Ks_ current. The drug significantly decreased the amplitudes of the I_NaL_ and I_CaL_ currents at 30 µM. Hesperetin (10 and 30 µM) did not change the action potential duration in normal preparations, however, in preparations where the repolarization reserve had been previously attenuated by 100 nM dofetilide and 1 µg/ml veratrine, caused a moderate but significant prolongation of repolarization. These results suggest that hesperetin at close to relevant concentrations inhibits the I_Ks_ outward potassium current and thereby reduces repolarization reserve. This effect in certain specific situations may prolong the QT interval and consequently may enhance proarrhythmic risk.

## Introduction

Citrus flavonoids like hesperetin can be found in large quantities in lemon, orange and grapefruit^1,2^. It is generally considered that they have beneficial effects. These beneficial effects include their well-documented antioxidant^3^ and antidiabetic^4^ effects. Epidemiological evidence also indicates reduced incidence of coronary heart disease and related mortality due to the consumption of these fruits^5^. Other epidemiological studies show protective effects of citrus alkaloids against ischemic stroke^6,7^ and cancer^8^. This latter observation is also supported by experimental data^9^.

However, in some clinical studies cardiac repolarization instability was also reported following orange and grapefruit juice consumption in healthy human volunteers who were not taking any medications^10^, suggesting that in certain sporadic situations and cases, this may enhance arrhythmia susceptibility.

In vitro studies indicated that hesperetin and naringenin as aglycone metabolites of hesperidin and naringin reduced hERG potassium current in Xenophus oocytes^10,11^. It is generally considered that hERG current and consequent QT_c_ prolongation increase the risk of Torsades de Pointes (TdP) cardiac arrhythmia and consequently, sudden cardiac death^12^. Although drug induced QT_c_ prolongation and TdP development is rare in general, however, its incidence can be increased in certain individuals with impaired repolarization reserve^13–15^.

Since hesperidin/hesperetin are the main flavonoids in orange juice^2,11^, which is one of the most frequently consumed beverages in many countries, in this study we investigated the cardiac electrophysiological effects of hesperetin in rabbit and dog cardiac ventricular preparations with intact and with reduced repolarization reserve.

## Results

The effects of hesperetin on several important ionic currents were studied in rabbit ventricular myocytes using the manual patch-clamp technique. However, the kinetic properties of rabbit I_to_ current are very different from that of human I_to_ and the conducting channels are also different: rabbit I_to_ is conducted by Kv 1.4 but in human mostly by Kv 4.3 channels. Therefore, I_to_—and also I_K1_—measurements were performed in dog ventricular myocytes because dog I_to_—similar to that in human—is also conducted by Kv 4.3 channels.

**Figure 1:**
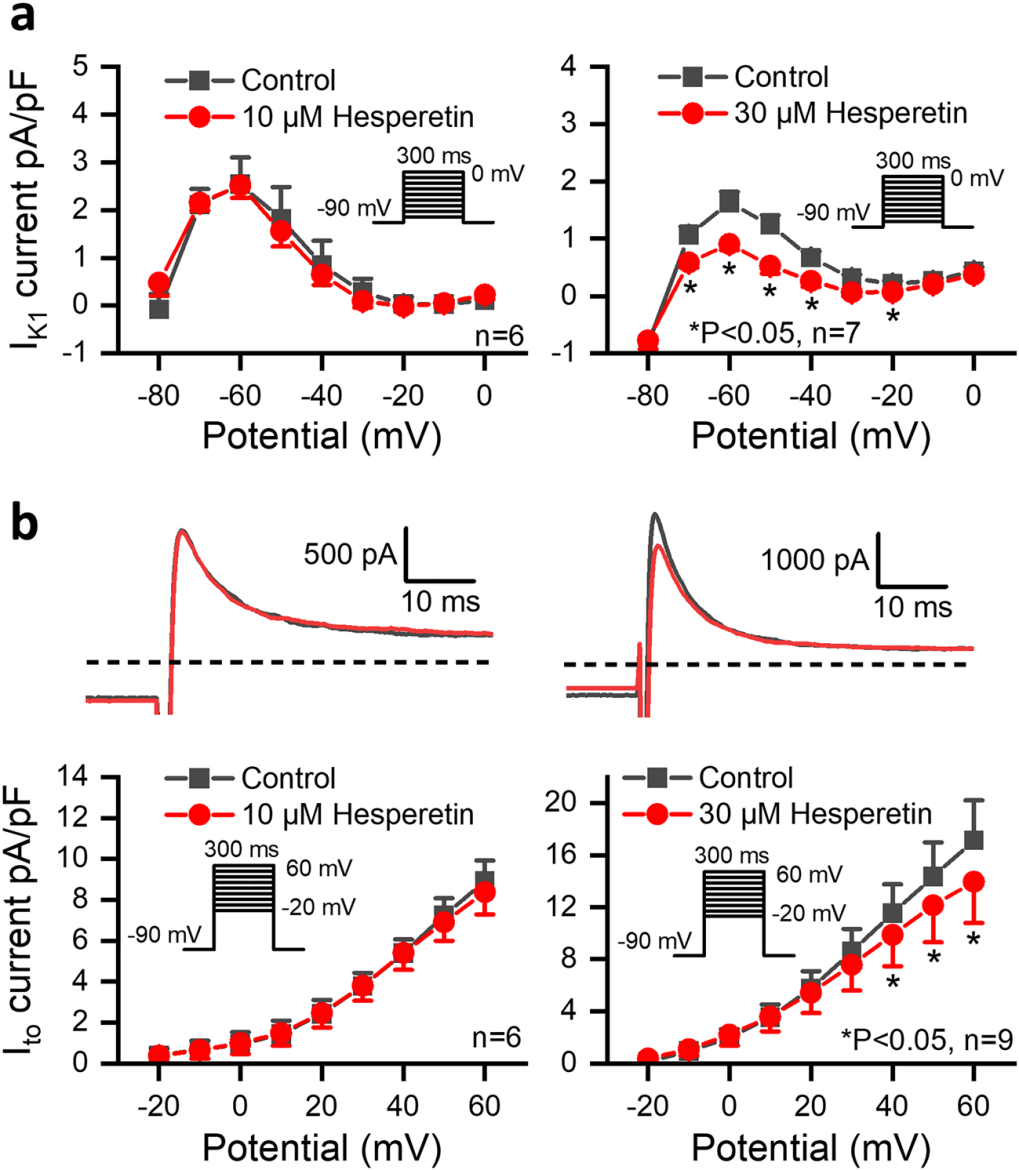
Effect of hesperetin on the inward rectifier (I_K1_) and transient outward (I_to_) potassium currents in dog left ventricular myocytes. (**a**) shows the effect of 10 µM and 30 µM hesperetin on I_K1_ current. (**b**) indicates original I_to_ current recordings (*top*) and current–voltage curves for I_to_ (*bottom*) in control conditions and after administration of 10 µM and 30 µM hesperetin. The insets show the applied voltage protocols. The dashed lines indicate zero current levels. Data are expressed as means ± SEM.

The effect of hesperetin on I_K1_ was studied in dog ventricular myocytes in 10- and 30 µM concentrations using the manual patch-clamp technique. Voltage pulses of 300 ms durations between − 80 and 0 mV with a pulsing cycle length of 3 s were applied from − 80 mV holding potential and the steady state current at the end of the voltage pulses was determined. As Fig. 1a indicates, the I_K1_ current was not significantly affected by 10 µM hesperetin, however, 30 µM hesperetin significantly reduced I_K1_ amplitude (by 41.6 ± 7.5% at − 60 mV, n = 7).

The effect of 10 and 30 µM hesperetin on the I_to_ current was studied also in dog ventricular myocytes with 300 ms test pulses from − 80 mV holding potential between 0 and 60 mV with a pulse cycle length of 3 s. The difference between the peak outward and current at the end of the test pulse was determined as the I_to_ current. Figure 1b shows that 10 µM hesperetin did not change, however, 30 µM hesperetin significantly decreased the amplitude of I_to_ (by 20.7 ± 5.1% at 50 mV, n = 9).

**Figure 2:**
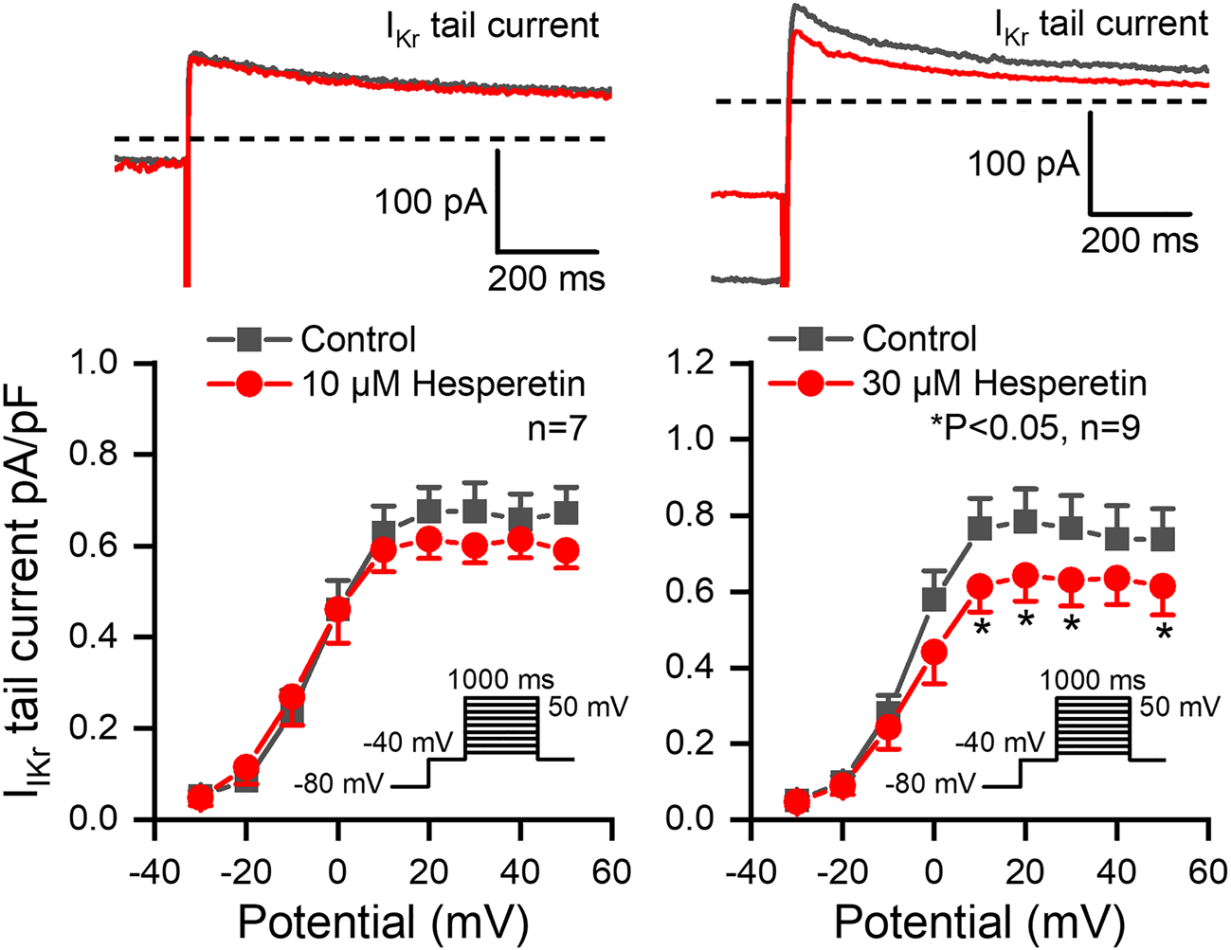
Effect of hesperetin on the rapid delayed rectifier potassium current (I_Kr_) in rabbit left ventricular myocytes. The panels display the tail current section of original I_Kr_ current traces (*top*) and current–voltage curves for I_Kr_ in control conditions and in the presence of 10 µM and 30 µM hesperetin. The insets show the applied voltage protocols. The dashed lines refer to the baseline for I_Kr_ tail current level after the test pulse at -40 mV. Data are expressed as means ± SEM.

The I_Kr_ current was measured in rabbit ventricular myocytes with test pulses of 1 s duration between − 30 mV and 50 mV at 20 s cycle length. The holding potential was − 80 mV, and during measurements a 500 ms long prepulse to − 40 mV was applied in order to ensure the baseline current. The amplitude of the deactivating tail current after the end of the test pulse at − 40 mV was measured as the I_Kr_ current. Figure 2 shows that 10 µM hesperetin did not, however, 30 µM hesperetin significantly reduced the amplitude of I_Kr_ (by 16.4 ± 7.7% at 50 mV, n = 9).

**Figure 3:**
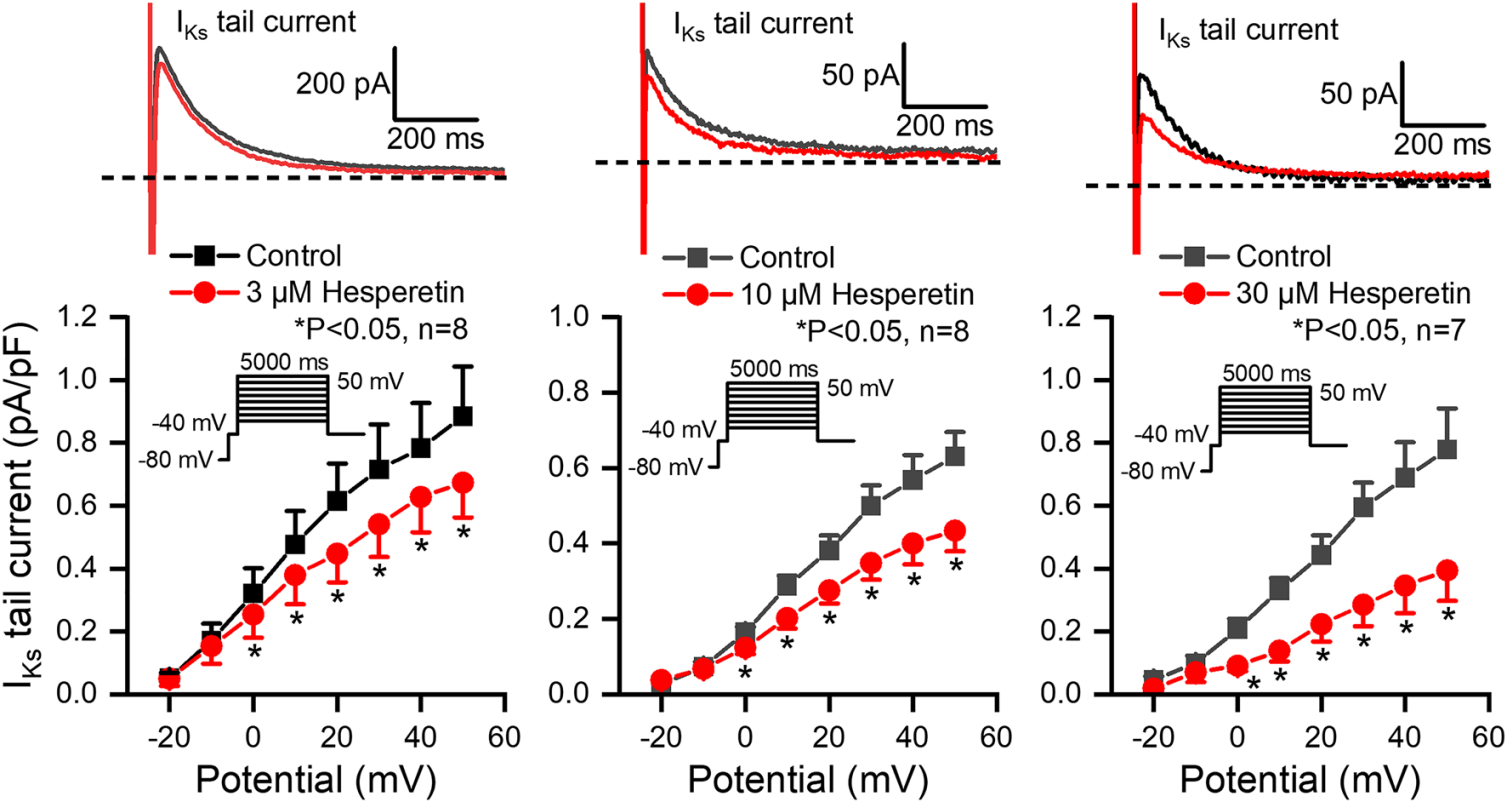
Effect of hesperetin on the slow delayed rectifier potassium current (I_Ks_) in rabbit left ventricular myocytes. The panels indicate the tail current section of original I_Ks_ current traces (*top*) and current–voltage curves for I_Ks_ in control conditions and in the presence of 3 µM, 10 µM and 30 µM hesperetin. The insets show the applied voltage protocols. The dashed lines refer to the baseline for I_Ks_ tail current level after the test pulse at -40 mV. Data are expressed as means ± SEM.

The I_Ks_ current was measured in rabbit ventricular myocytes with test pulses of 5 s duration between − 30 and 50 mV at pulse cycle length of 10 s. The holding potential was − 80 mV. The amplitude of the deactivating tail current after the end of the test pulse at − 40 mV was determined as I_Ks_. Figure 3 shows that hesperetin at 3, 10 and 30 µM concentrations significantly and in a concentration-dependent manner decreased the I_Ks_ tail current (by 20.6 ± 4.3%, n = 8; by 31.9 ± 4.0%, n = 8; by 52.0 ± 5.4%, n = 7; at 50 mV).

**Figure 4:**
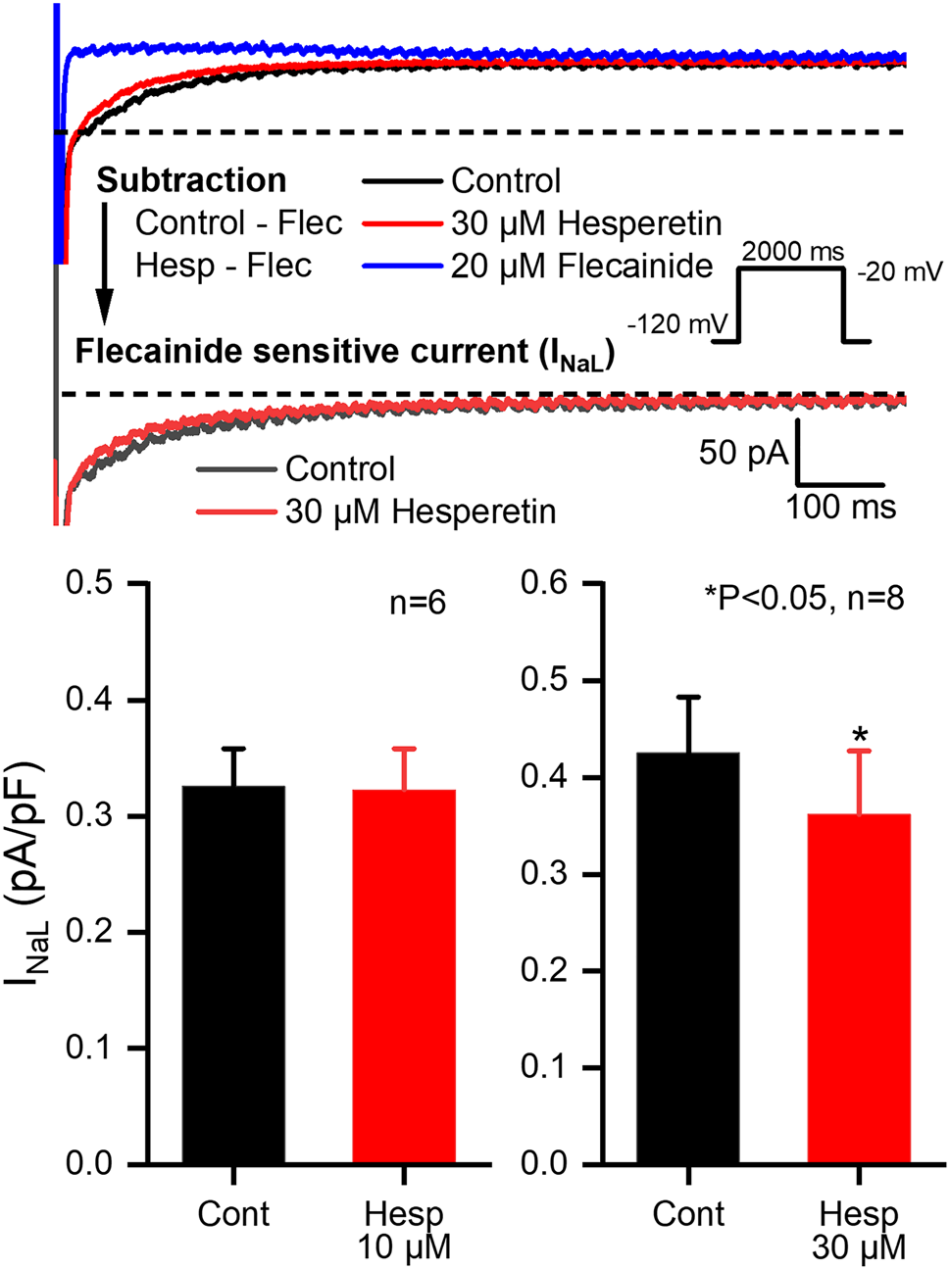
Effect of hesperetin on the late sodium current (I_NaL_) in rabbit left ventricular myocytes. Effects of 10 µM and 30 µM hesperetin administration on I_NaL_ current are indicated showing original current recordings (*top*) and bar diagrams (*bottom*). The inset shows the applied voltage protocol. The dashed lines indicate zero current levels. Data are expressed as means ± SEM.

I_NaL_ was measured from − 120 mV holding potential by applying 2000 ms long test pulses to − 20 mV with a pulse cycle length of 5 s in rabbit ventricular myocytes. After control recordings, hesperetin was applied for 5–7 min, followed by 20 µM flecainide in the presence of hesperetin in order to fully block I_NaL_ and the difference currents measured in control, after hesperetin and after flecainide application were determined. The amplitude of the currents at 50 ms of the test pulses compared to the steady state current at the end of the pulse were designated as I_NaL_ in order to avoid possible interference of improper voltage control due to the very rapidly activating and large fast I_Na_^16^. As Fig. 4 shows, 30 µM hesperetin moderately and significantly decreased I_NaL_ in rabbit ventricular myocytes (by 16.8 ± 5.6%, n = 8).

**Figure 5:**
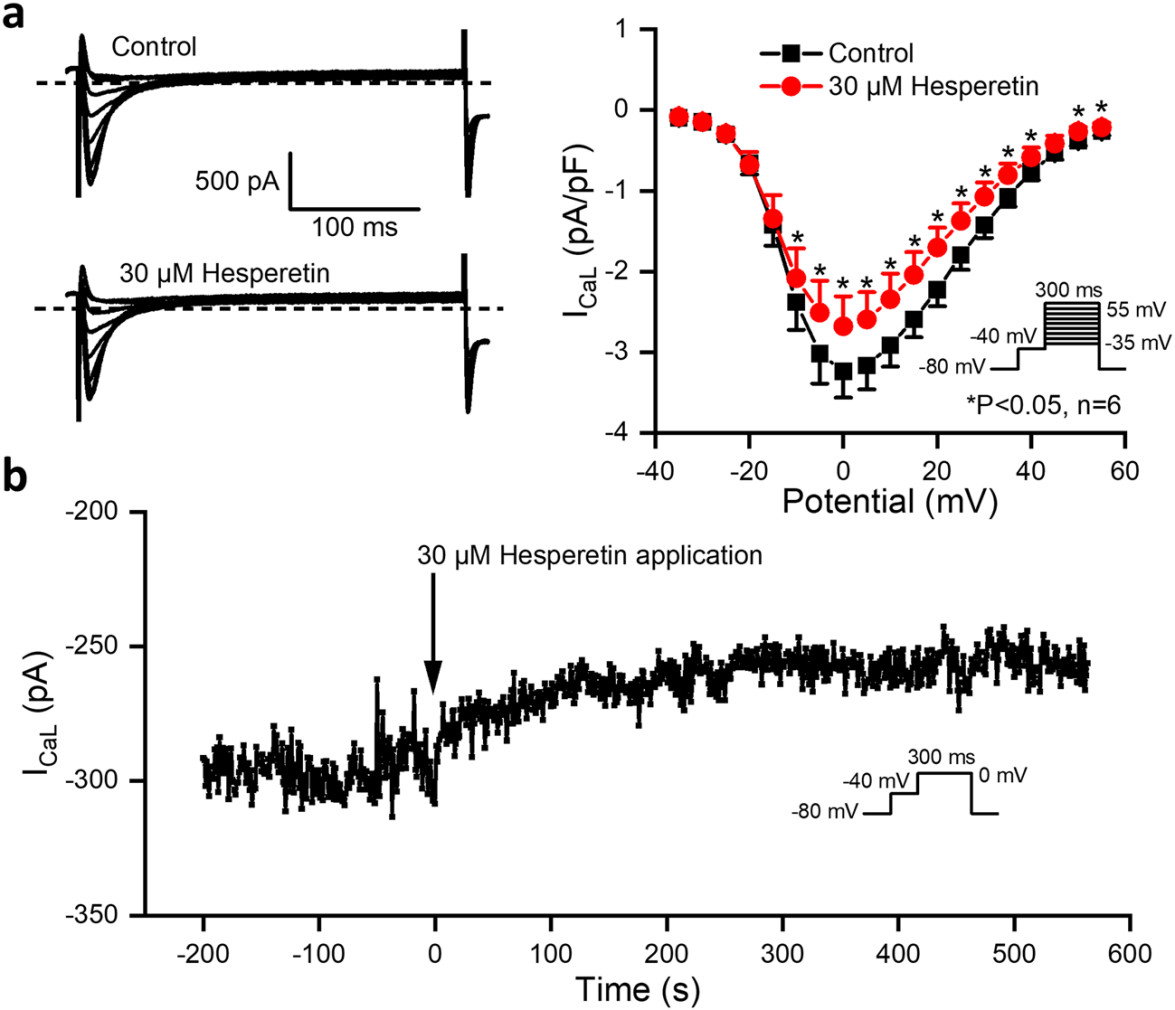
Effect of hesperetin on the L-type calcium current (I_CaL_) in rabbit left ventricular myocytes. Upper panels (**a**) indicate original current records (*left*) and current–voltage relationship (*right*) of I_CaL_ recorded with 1000 ms pulsing cycle length in control conditions and in the presence of 30 µM hesperetin. Bottom panel (**b**) displays the effect of hesperetin in a representative experiment on I_CaL_ current as a function of time. Negative numbers in the time scale indicate control periods, while the positive time range shows I_CaL_ current level after application of 30 µM hesperetin. The pulse cycle length was 1000 ms. The insets show the applied voltage protocols. The dashed lines indicate zero current levels. Data are expressed as means ± SEM.

I_CaL_ was measured in rabbit ventricular myocytes with 300 ms long voltage pulses between − 35 to 55 mV from a holding potential of − 80 mV with a 20 ms long − 40 mV prepulse by applying it with a pulse cycle length of 1 s. As Fig. 5 shows, hesperetin at 30 µM moderately but significantly decreased the amplitude of I_CaL_ (by 18.3 ± 5.4% at 0 mV, n = 6).

**Figure 6:**
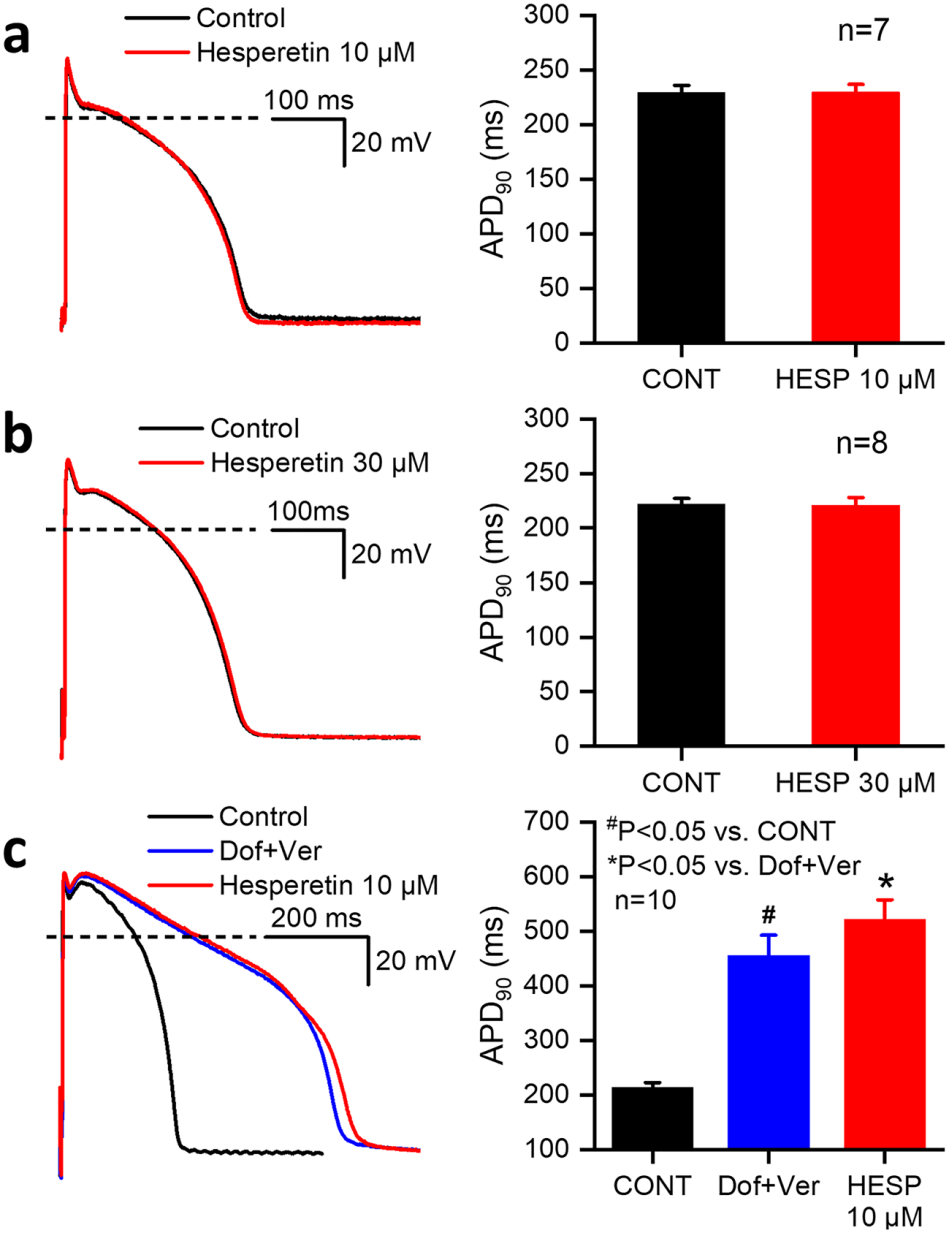
Effect of hesperetin on the action potential duration (APD_90_) in dog right ventricular papillary muscle preparations. (**a**) and (**b**) Displays representative action potential waveforms and bar diagrams in control conditions and after administration of 10 µM and 30 µM hesperetin, respectively. Bar diagram in (**c**) shows the effect of 10 µM hesperetin on the action potential duration when repolarization was previously prolonged by 500 nM dofetilide and 1 µg/ml veratrin. The dashed lines indicate zero potential levels. Data are expressed as means ± SEM.

Cardiac action potentials were measured by the conventional microelectrode technique in dog right papillary muscle preparations at the stimulation cycle length of 1 s. As Fig. 6a and b indicate, hesperetin (10 or 30 µM) did not change significantly the action potential waveform and action potential duration after 30–40 min of hesperetin superfusion. The effects of hesperetin on action potential repolarization in preparations with impaired repolarization reserve were also investigated. Application of 500 nM dofetilide and 1 µg/ml veratrine greatly attenuates repolarization reserve since dofetilide completely blocks I_Kr_ at this concentration, while veratrine delays the inactivation of I_NaL_ increasing this inward current in the plateau phase of the action potential. In these preparations with impaired repolarization reserve, when consequently repolarization was prolonged, application of 10 µM hesperetin moderately and further, significantly lengthened action potential duration (Fig. 6c) (by 14.0 ± 3.6% vs. dofetilide + veratrine, n = 10). To avoid possible interference with slow and variable development of APD prolongation by dofetilide + veratrine, hesperetin was applied at the time when APD reached steady-state for at least 30 min.

## Discussion

The main new findings in this study are the following: (i) hesperetin—an important citrus flavonoid—decreases the magnitude of I_Ks_ at physiologically relevant or slightly higher concentrations; (ii) at higher concentrations, hesperetin inhibits both multiple outward potassium, such as I_Kr_, I_K1_, I_to_, I_Ks_, and inward I_CaL_ and I_NaL_ currents in rabbit and dog cardiac ventricular myocytes; (iii) normal cardiac action potential duration was not significantly or only minimally affected by hesperetin, however, when repolarization reserve was reduced, hesperetin evoked moderate but significant further prolongation of ventricular action potential in the dog heart.

In this study, we investigated hesperetin in two concentrations. The lower concentrations (3–10 µM) correspond with the upper plasma concentration limit observed after the ingestion of relatively large quantities of orange^17,18^ or grapefruit^17,18^ or their juices. The high concentration (30 µM) can be considered too high to be observed normally.

The effect of hesperetin on native cardiac ventricular transmembrane ionic currents has not been studied yet. In Xenopus oocyte and in HEK293 cell line it was reported that relatively high concentrations (IC_50_ = 288.8 µM and 23.1 µM) of hesperetin inhibited hERG channel^10,11^ and Kv 1.5 channel^19^ which are the pore-forming channel subunits of the rapid (I_Kr_) and ultrarapid (I_Kur_) delayed rectifier potassium currents, respectively. Although I_Kur_ was described in dog ventricular myocytes^20^, its functional role in ventricular repolarization is still unclear. Naringenin and hesperetin induced hERG current inhibition was suggested as a possible explanation for the reported QT_c_ prolongation in humans following grapefruit juice ingestion^10^. However, hERG channel inhibition can give both false positive, and its absence false negative prediction for native I_Kr_ and action potential duration changes^21^. Therefore, effects of compounds such as hesperetin should be also investigated for their effects on native currents and action potentials as well. In HEK 293 cell line and in human atrial myocytes it was reported that hesperetin decreased Nav 1.5 and I_Na_ currents at high concentrations with IC_50_ of 63 µM^22^. In rat ventricular myocytes and LQT3 mutant Nav 1.5 expressed channels, both peak and slowly inactivating I_Na_ were depressed by hesperetin with IC_50_ ranging from 31 to 55.6 µM^23^. In this report, in Langendorff-perfused rat hearts QRS widening and QT_c_ shortening were also observed with hesperetin at concentrations of 30 and 100 µM. These latter results are in good agreement with our experimental findings with I_NaL_ in rabbit ventricular myocytes, since I_NaL_ contributes to the repolarization during the plateau phase and its inhibition by hesperetin can limit APD prolongation by the compound explaining the lack of action potential duration prolongation after hesperetin administration.

In spite of the overwhelming evidence suggesting health benefits due to consumption of citrus fruits and juices it was also reported that grapefruit juice delayed cardiac repolarization expressed as QT_c_ interval prolongation in healthy volunteers^10^. Subsequent later studies confirmed this observation in both healthy subjects and in long QT patients^15^, as well as in cardiomyopathic^24^ patients. The temporal dispersion of cardiac repolarization, measured as the short term variability of the QT interval, was also enhanced in these studies, suggesting increased susceptibility to cardiac arrhythmias. In a recent case study^13^, it was reported that a 52-year-old man with a previously asymptomatic cLQTS type 1 experienced dizziness and high heart rate after ingestion of two glasses of orange juice. His ECG recording showed an unusually long QT_c_ of 638 ms. After discharge from the hospital, on the sixth day his QT_c_ on the ECG decreased to 524 ms and upon his six-month follow-up visit with no citrus juice consumption, his QT_c_ duration was 476 ms. Allopurinol 300 mg once daily and bisoprolol 2.5 mg twice daily were the only chronic medications taken by the patient.

The effect of hesperetin on the ventricular action potential can be explained by its modulation of transmembrane ionic currents. At low (3–10 µM) relevant concentrations (at concentrations corresponding with plasma levels measured in humans following juice consumption), hesperetin modestly but significantly decreases the amplitude of I_Ks_. This effect on the I_Ks_ does not significantly change action potential duration since in the normal heart the action potential plateau duration is relatively short (less than 200 ms), and due to the slow activation kinetics of I_Ks_ at the plateau voltage, little I_Ks_ can be activated. Consequently, inhibition of I_Ks_ does not result in significant changes of the configuration of the normal action potential. However, in preparations where repolarization has been previously prolonged by dofetilide and veratrine, I_Ks_ has more time to activate, therefore its contribution to repolarization is augmented. In addition, since in our experiments the repolarization reserve was attenuated by I_Kr_ block (dofetilide) and enhancement of I_NaL_ (veratrine), further effect on any other repolarizing current such as inhibition of I_Ks_ would have more impact on the repolarization process. Therefore, in this situation the inhibition of I_Ks_ by hesperetin can cause further prolongation of action potential duration. A similar effect with other I_Ks_ inhibitors was experimentally demonstrated and described earlier^25,26^ arguing for the importance of I_Ks_ as part of the repolarization reserve^27–30^. Further increasing the concentration of hesperetin (30 µM), other potassium currents were also affected due to the complex inhibitory effects of the drug on inward currents. Thus, inhibiting outward currents would prolong repolarization with increasing concentrations but when the concentration is increased even further, the inhibition of inward currents would result in the opposite effect, i.e. in shortening of repolarization. Therefore, the net effect on repolarization is minimalized since it is likely that these currents can counterbalance each other resulting in very small or not significant effects on the action potential duration. The net effect, therefore, is consistent with the observation that hesperetin does not cause frequent proarrhythmic complications in the population. The reported plasma concentration of hesperetin in human is in the low micromolar range^17,18^, therefore, hesperetin concentrations above 10 µM should have limited significance. In addition, it is often difficult to compare effects after in vitro and in vivo applications. Also, after orange or grapefruit ingestion other flavonoids like naringenin also appear in the blood and have been reported to inhibit hERG current^10,18,31^.

Importantly, since natural orange juice has high potassium content^32,33^, its ingestion can increase plasma potassium levels, thus opposing QT prolongation and acting as a possible “protective” mechanism.

Although in this study we provided evidence that hesperetin which is an important flavonoid and abundantly present in orange, lemon and grapefruit, inhibits various cardiac potassium currents and lengthens repolarization in the setting of reduced repolarization reserve, we do not suggest that consumption of citrus fruit and their juices are unhealthy or dangerous. Overwhelming evidence indicates their beneficial effects preventing various diseases, therefore, consumption of citrus fruits and their juices are an important part of a healthy diet. However, in pathological settings when repolarization reserve may be impaired, such as in long QT syndromes, heart failure, hyperthrophic cardiomyopathy or in elite athletes, consumption of large quantities of citrus juices may increase the risk of cardiac arrhythmia development. Therefore, in these settings particular attention and routine ECG screening would be useful to avoid possible complications.

The present study has several limitations. In our experiments, we applied rather high doses of hesperetin and we observed moderate effects which may lead to overestimation of the potentially increased proarrhythmic risk of hesperetin. Also, citrus fruits contain several other alkaloids and ingredients which can modify the outcome compared to that seen after only hesperetin administration. Our experiments were carried out in healthy normal cardiac preparations in the absence of beta-adrenergic stimulation that can yield different results than those isolated from diseased or structurally and electrically remodeled hearts or following robust sympathetic stimulation, since I_Ks_ is dependent on cAMP as it was demonstrated by Volders et al.^34,35^. Therefore, further experiments with hesperetin and/or other citrus alkaloids on diseased heart preparations would be necessary to properly elucidate the possible enhanced arrhythmogenic risk that may be associated with these flavonoids.

The results of this study suggest that the citrus flavonoid hesperetin at a relevant concentration inhibits I_Ks_ and at higher concentration several other cardiac potassium currents, thereby attenuating repolarization reserve. This effect, in addition to its well documented beneficial effects, in certain specific pathological situations may prolong QT_c_ interval and may consequently enhance proarrhythmic risk.

## Methods

### General methods

Animal maintenance and research were conducted in accordance with the National Institutes of Health Guide for the Care and Use of Laboratory Animals. All procedures involving animals were approved by the Ethical Committee for the Protection of Animals in Research of the University of Szeged, Szeged, Hungary (approval numbers: I-74-15-2017 and I-74-24-2017) and by the Department of Animal Health and Food Control of the Ministry of Agriculture and Rural Development (authority approval numbers: XIII/3330/2017 and XIII/3331/2017) and conformed to the rules and principles of the 2010/63/EU Directive. The animals were purchased from an experimental animal breeder, Ásotthalom, Hungary (breeder’s authority approval number: XXXV/2018) certified by the Department of Animal Health and Food Control of the Ministry of Agriculture and Rural Development, Hungary. All experiments are reported in compliance with the ARRIVE guidelines.

### Conventional microelectrode technique

Action potentials were recorded in right ventricular papillary or trabecular muscle preparations obtained from the hearts of dogs using the conventional microelectrode technique previously described in detail^36^. Briefly, the preparations were mounted in a tissue chamber of 50 ml volume individually. The experiments were performed using a modified Locke’s solution containing (in mM): NaCl 128.3, KCl 4, CaCl_2_ 1.8, MgCl_2_ 0.42, NaHCO_3_ 21.4, and glucose 10. The pH of this solution was set between 7.35 and 7.4 when gassed with 95% O_2_ and 5% CO_2_ at 37 °C. Each preparation was stimulated through a pair of platinum electrodes in contact with the preparation at a constant basic cycle length of 1000 ms. Transmembrane potentials were recorded by an Experimetria high impedance amplifier (Experimetria, type 309, Budapest, Hungary) and data acquisition and stimulation were performed with an in-house developed software (APES) following a 60-min equilibrium time after mounting the preparations using conventional glass microelectrodes, filled with 3 M KCl.

The measurements where the resting membrane potential of the recorded action potential was more positive than − 70 mV and/or the action potential amplitude was less than 90 mV, were excluded from the analyses.

### Patch-clamp measurements

Ventricular myocytes from New Zeeland rabbits and Beagle dogs were enzymatically dissociated as described in detail previously^36,37^. A single droplet of cell suspension was placed in a transparent recording chamber mounted on the stage of an inverted microscope (Olympus IX51, Olympus, Tokyo, Japan), and individual myocytes were allowed to settle and adhere to the chamber bottom for at least 5–10 min before superfusion was initiated and maintained by gravity. Only rod-shaped cells with clear striations were used. HEPES-buffered Tyrode’s solution (composition in mM: NaCl 144, NaH_2_PO_4_ 0.4, KCl 4.0, CaCl_2_ 1.8, MgSO_4_ 0.53, glucose 5.5 and HEPES 5.0, at pH of 7.4) served as the normal superfusate.

Micropipettes were fabricated from borosilicate glass capillaries (Science Products GmbH, Hofheim, Germany), using a P-97 Flaming/Brown micropipette puller (Sutter Co, Novato, CA, USA), and had a resistance of 2–3 MOhm when filled with pipette solution. The membrane currents were recorded with Axopatch-200B amplifiers (Molecular Devices, Sunnyvale, CA, USA) by means of the whole-cell configuration of the patch-clamp technique. The membrane currents were digitized with 250 kHz analogue-to-digital converters (Digidata 1440A, Molecular Devices, Sunnyvale, CA, USA) under software control (pClamp 10, Molecular Devices, Sunnyvale, CA, USA). All patch-clamp experiments were carried out at 37 °C.

#### Measurement of L-type calcium current

The L-type calcium current (I_CaL_) was recorded in HEPES-buffered Tyrode’s solution supplemented with 3 mM 4-aminopyridine. A special solution was used to fill the micropipettes (composition in mM: CsCl 125, TEACl 20, MgATP 5, EGTA 10, HEPES 10, pH was adjusted to 7.2 by CsOH).

#### Measurement of potassium currents

The inward rectifier (I_K1_), the transient outward (I_to_), the rapid (I_Kr_) and the slow (I_Ks_) delayed rectifier potassium currents were recorded in HEPES-buffered Tyrode’s solution. The composition of the pipette solution (mM) was: KOH 110, KCl 40, K_2_ATP 5, MgCl_2_ 5, EGTA 5, HEPES 10 (pH was adjusted to 7.2 by aspartic acid). 1 µM nisoldipine was added to the bath solution to block I_CaL_. When I_Kr_ was recorded, I_Ks_ was inhibited by using the selective I_Ks_ blocker HMR-1556 (0.5 µM). For I_Ks_ measurements, I_Kr_ was blocked by 0.1 µM dofetilide and the bath solution contained 0.1 µM forskolin.

#### Measurement of late sodium current

The late sodium current (I_NaL_) was activated by depolarizing voltage pulses of 2 s at -20 mV from holding potential of -120 mV with pulsing cycle lengths of 5 s. After incubation with the drug for 5–7 min, the external solution was replaced with a solution containing 20 µM flecainide, this concentration completely blocked the I_NaL_. The external solution was HEPES-buffered Tyrode’s solution supplemented with 1 µM nisoldipine, 0.5 µM HMR-1556 and 0.1 µM dofetilide in order to block I_CaL_, I_Ks_ and I_Kr_ currents. The composition of the pipette solution (in mM) was: CsCl 125, TEACl 20, MgATP 5, EGTA 10, HEPES 10, pH was adjusted to 7.2 by CsOH).

### Statistics

Microsoft Excel (Microsoft Office Professional Plus 2016) and Origin software (2021b, OriginLab) packages were used for statistical analysis. Data were expressed as mean ± standard error of the mean (S.E.M.). Paired Student's t-test was applied to evaluate whether there was a statistically significant difference between the means in the self-control experiments. Data were considered statistically significant when *p* < 0.05.

## Data Availability

The data that support the findings of this study are available from the corresponding author upon reasonable request.
